# A case of parosteal osteosarcoma with a rare complication of myositis ossificans

**DOI:** 10.1186/1477-7819-10-260

**Published:** 2012-11-29

**Authors:** Maria Silvia Spinelli, Carlo Perisano, Carlo Della Rocca, Jendrick Hardes, Carlo Barone, Carlo Fabbriciani, Giulio Maccauro

**Affiliations:** 1 Department of Orthopedics and Traumatology, Catholic University Hospital “Agostino Gemelli”, L.go A. Gemelli, 1-00168 Rome, Italy; 2 Department of Experimental Medicine, Sapienza University of Rome, Polo Pontino, I.C.O.T, Latina, Italy; 3 Department of Orthopedics and Tumor Orthopedics, University Hospital, Muenster, Germany; 4 Department of Clinical Oncology, Catholic University Hospital, Rome, Italy

**Keywords:** Distal ulna resection, Parosteal osteosarcoma, Ulna, Ulna reconstruction

## Abstract

We report the case of a parosteal osteosarcoma of the distal ulna, treated with wide resection without reconstruction. The patient developed lung metastasis and a mass in the interosseus membrane of the forearm proximally to the osteotomy. The lung mass was found to be a metastasis from parosteal osteosarcoma and the biopsy of the forearm mass revealed a myositis ossificans. The suspicion of a recurrence of parosteal osteosarcoma, already metastatic, led to a second wide resection with no reconstruction. A slice of the radial cortex was taken during this second procedure. From a histological point of view, good margins were achieved and diagnosis of myositis ossificans was confirmed. Two months later, a radius fracture occurred and a synthesis, with plate and screws, as added with poly(methyl methacrylate) (PMMA) to reconstruct the bone loss, was performed. Indication of the reconstructive technique and the complication after distal ulna resection in oncologic surgery are discussed in this paper.

## Background

Parosteal osteosarcoma is the most common type of osteosarcoma originating from the cortex, accounting for 5% of all osteosarcomas [1]. Furthermore, the ulna is rarely affected by bone tumors.

Due to the rarity of the localization, distal ulna resection and reconstructive options after oncologic surgery are still debated. The literature provides conflicting choices, with reports advocating no reconstruction after resection [2,3], soft tissue stabilization procedures [4] and bone graft augmentation [5].

Given the lack of consensus in the literature on this rare condition, we present our clinical case in which parosteal osteosarcoma was treated without reconstruction, along with complications occurring after our surgical procedure.

## Case presentation

**Figure 1:**
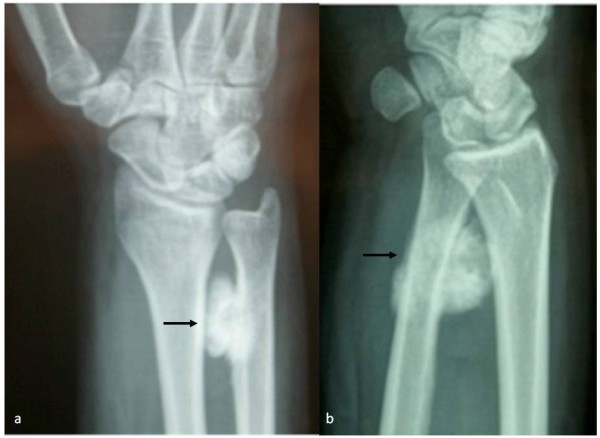
Antero-posterior (a) and lateral (b) view of wrist showing the ossifying mass surrounding the distal ulna.

**Figure 2:**
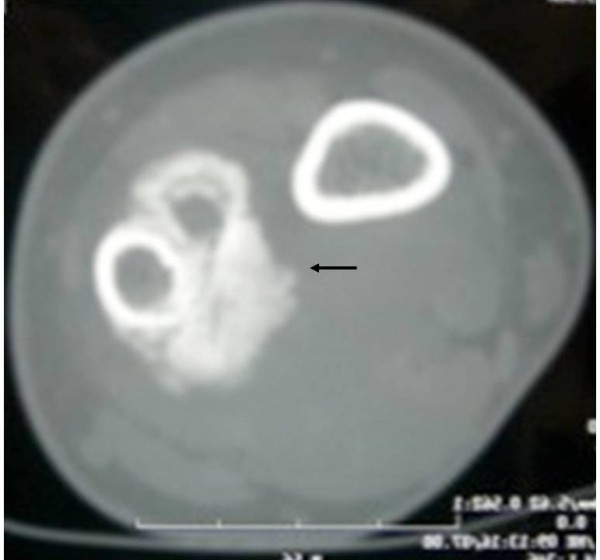
CT scans showing the ossifying mass arising from the cortex without significant bone marrow infiltration.

We report the case of a 41-year-old man who presented with a mass of one-year duration in the left (non-dominant) wrist. The mass was localized in the ulnar border, and was painful at rest (at night) and in flexion-extension and ulno-radial deviation of his wrist. X-rays detected a mass around the medial border of the distal ulna with periosteal reaction (Figure 1a, b). Magnetic resonance imaging (MRI) and computed tomography (CT) showed no marrow involvement and confirmed the bony mass was arising from the cortex, with a lower density than the ulnar bone (Figure 2).

**Figure 3:**
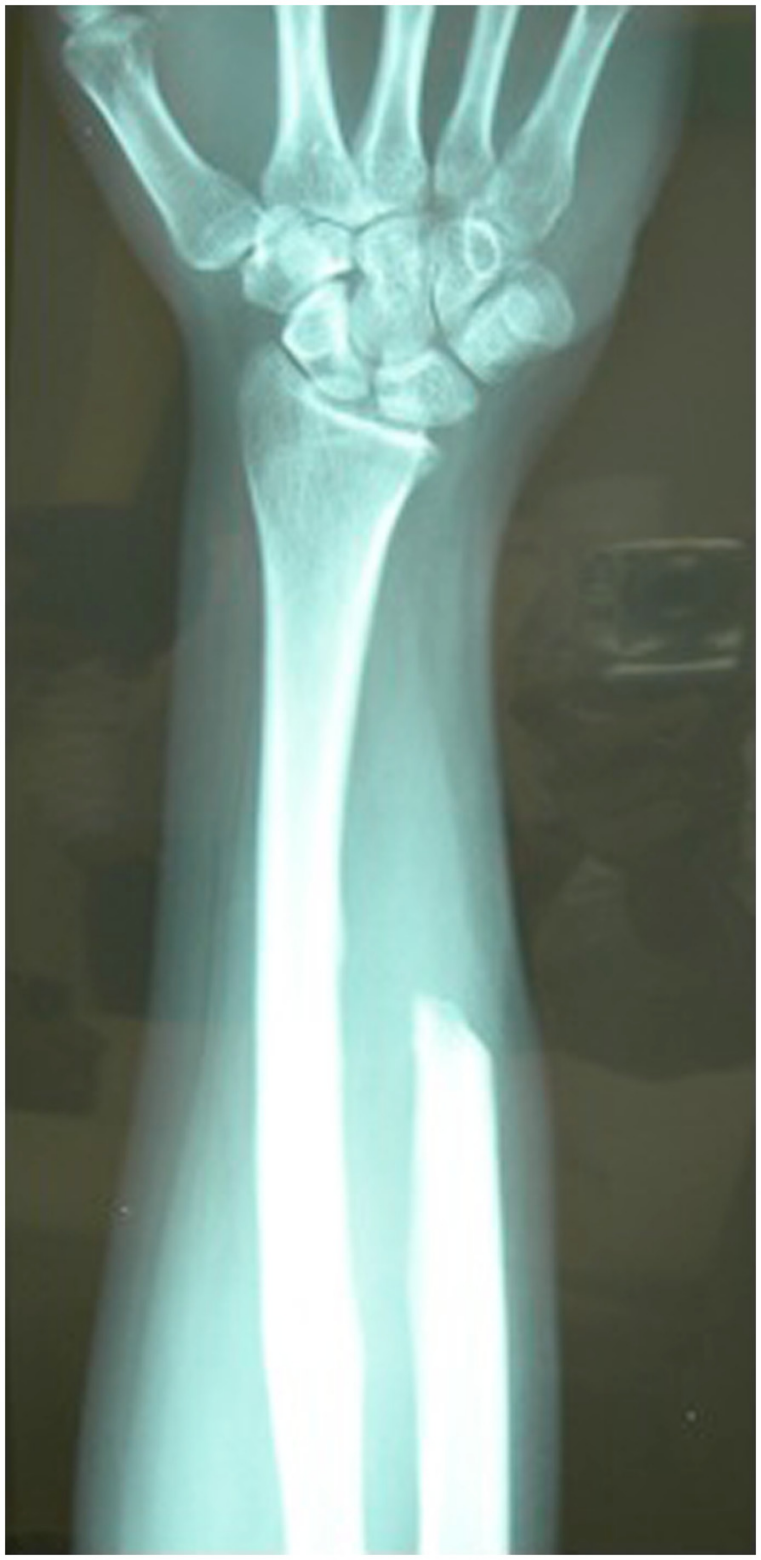
**X-rays after distal ulna resection. **No mass left in the forearm. The interosseus membrane seems to be free from other ossifying lesions.

Incisional biopsy was performed and the diagnosis was parosteal osteosacoma (Grade 3). Full oncologic evaluation identified a lung mass, smaller than 1 cm, in a chest CT that was not diagnosed as a metastasis (there was no metabolic activity in the body scan). Imaging follow-up (18 months, X-ray and CT scan) showed the mass was stable. The patient underwent an *en-bloc* resection of the distal ulna (15 cm from the ulno-carpal joint) with disarticulation of the ulno-carpal joint, performed by the orthopedic oncologic senior surgeon (Figure 3). Frozen sections of the interosseus membrane and proximal ulna stump marrow were negative for tumor cells. Good margins were achieved from a histological point of view and the diagnosis was confirmed. A cast was placed for four weeks. At follow-up, the patient showed an excellent functional outcome.

He did not change his job (mild to heavy activity), nor his hours of work.

**Figure 4:**
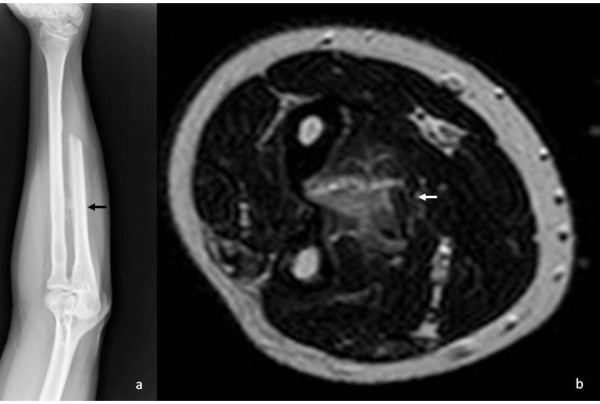
**After 18 months X-rays showed small ossifying mass in the interosseus membrane. **It is not clear if there was contact with the bone (**a**); MRI confirmed the presence of the mass that did not seem to involve the ulna or the radius (**b**).

**Figure 5:**
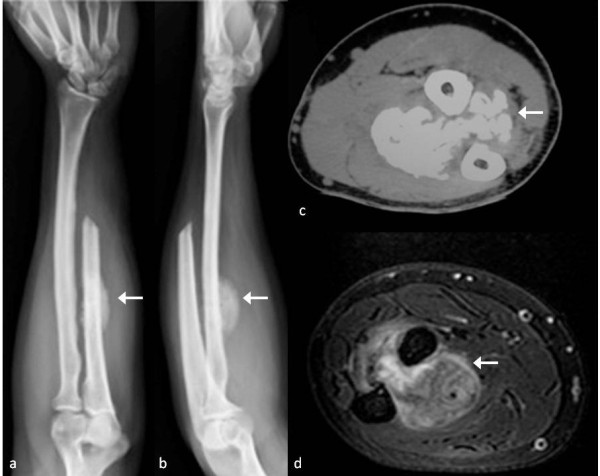
X-rays (a, b), CT scan (c) and MRI (d) show increased dimensions of the lesion but no contact with the bones after a few weeks.

**Figure 6:**
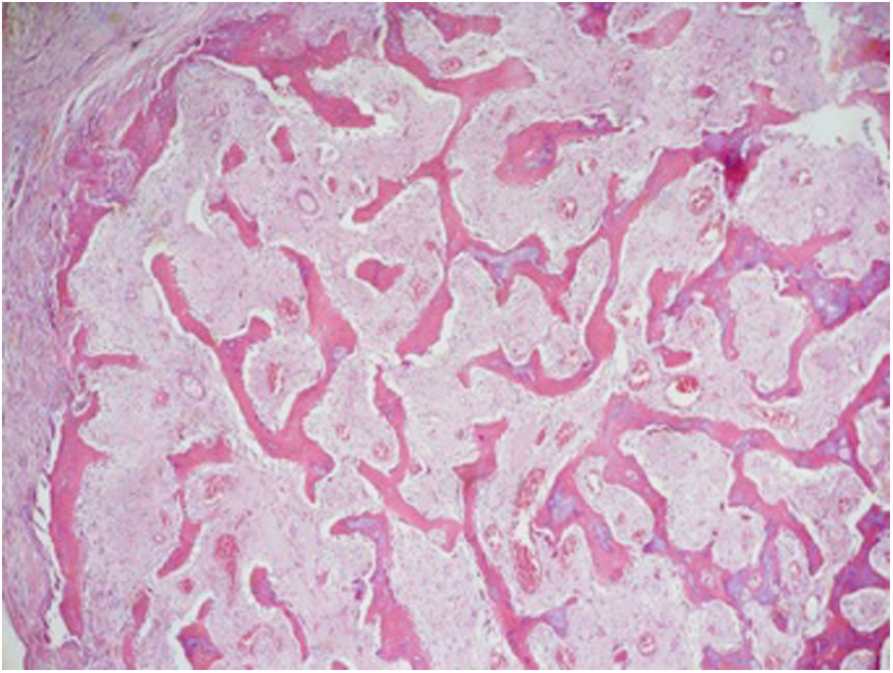
Myositis ossificans low magnification showing mature shell of bone at the periphery and bone trabecule.

At one-year follow-up, the patient had full range of motion, no sign of local recurrence, no pain during daily activities and no sign of instability, with only a mild clicking during pronation that did not bother him significantly. Radiographs showed no signs of instability. Fifteen months after surgery, the new forearm X-ray showed a small round mass (diameter <1cm), which was localized about 5 cm proximally from the ulnar resection site in the interosseous membrane (Figure 4a, b). The mass showed a rapid increase in dimension, reaching 5 × 3 cm in one month. The CT showed the mass had no contact with the ulna nor with the radius (Figure 5a-d). Since we suspected a recurrence of the parosteal osteosarcoma, we performed an incisional biopsy. The histological examination detected a myositis ossificans (Figure 6). In the same period, the patient underwent a lung mass resection because of the suspicion of a metastatic lesion from the parosteal osteosarcoma that was confirmed by the histological examination. The patient underwent chemotherapy because of the systemic disease. The orthopedic surgeon consequently decided to perform an *en-bloc* resection to remove the mass in the interosseous membrane with subsequent shortening of the ulna, leaving 5 cm of the proximal ulna. The ulnar nerve had to be removed during resection. Additionally, a lateral slice of the radius, close to the mass, was resected (less than one-fifth of the cortex). Histological examination confirmed myositis ossificans.

**Figure 7:**
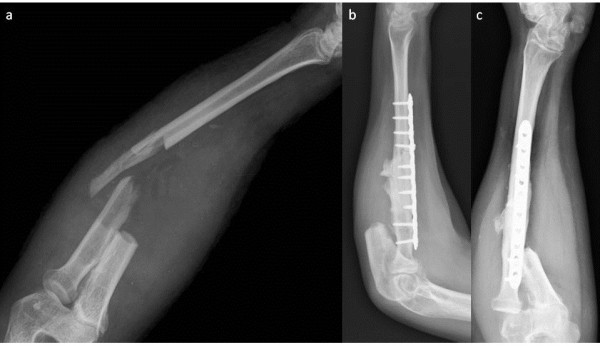
X-ray image of radius fracture (a) and the radius osteosynthesis with plate, screws and PMMA (b, c).

No stabilization technique for the proximal stump was performed. After two months, an accidental fracture affected the radius and an osteosynthesis with plate, screws and PMMA was subsequently performed to fill the bony defect (Figure 7a-c), according to Pezzilo *et al.*[6].

One year after the last surgical procedure, there was no local recurrence of the tumor or systemic or lung metastasis. The patient had complete ulnar nerve impairment, partial radial nerve deficit (only in the motor component), but the median nerve activity was normal.

During flexion the patient presented a loss of ulnar flexion of 20° and dorsal subluxation of the stump, which was mildly bothering him. He changed his job, but he reported no pain during his daily activities.

## Conclusions

The therapy for parosteal osteosarcoma is a large *en-bloc* resection. Inadequate excision of the tumor leads to a recurrence rate of 80% to 100%. While resection of the ulna is often relatively easy to perform, problems arise in the reconstruction of the defect.

Darrach’s procedure (resection of 1 to 2 cm of the distal ulna) for post traumatic and degenerative conditions is well described; however, patients undergoing oncological resection are different from the former because they are usually younger and highly demanding and reaching good margins for a longer bone resection leads to increased instability of the ulnar stump with dynamic radio-ulnar convergence. Clinically, this condition may lead to pain, weakness, loss of grip strength and dorsal subluxation of the distal ulnar stump, up to digital extensor tendon rupture and radio-carpal instability with ulnar translation of the carpus.

The literature describes good results from the use of different reconstruction procedures in order to minimize complications after wide excision of the distal ulna [7].

On the other hand, some authors suggest that in oncological resection of the distal ulna, reconstruction does not improve functional outcomes, and simple resection does not increase the complication rate caused by reconstructive techniques [2,3,8]. Although there are no data on the maximal length of distal ulna resection achievable without functional impairment, we deem that the length of the distal ulna resection, without reconstruction implying any functional impairment, would be no more than one-third of the entire bone.

A complication of distal ulnar resection, which is not mentioned in the literature to the best of the authors’ knowledge, is myositis ossificans arising from the interosseous membrane. We believe that the micro instability of the distal ulnar stump would be the cause of bleeding and consequent myositis ossificans in our case. This has led to difficulties in differential diagnosis with parosteal osteosarcoma.

On radiological examination, the lesion of myositis ossificans should not surround the host bone, and shows a transparent line that indicates the complete separation from the cortex (zonal sign). However, the location of the myositis, the simultaneous detection of lung metastasis from the parosteal osteosarcoma and the rapid increase of the mass raised the suspicion of recurrence from the primary tumor, and justified the second *en bloc* resection, even with a histological diagnosis of a benign lesion.

The management of this complication led to a proximal ulnar resection. The lack of reconstruction in this resection entails more complications. The patient complained of a dorsal subluxation of the ulnar remnant during elbow flexion.

After proximal ulna resection, the radius is the only bone which supports moment forces in movements and this probably led to a fracture without a trauma, even if the resection of the cortex was less than one-fifth of the entire diameter. We suggest, in the case of proximal ulnar resection, a preventive osteosynthesis of the radius to give a better mechanical stability to this segment and to avoid a possible fracture due to high moment forces on the bone segment.

We suggest that the patient undergoes an autograft reconstruction with a vascularized fibular graft. Other biological reconstructive options could be autografts, allografts or a non-biological spacer made with Stainmann and cement to avoid gross instability. Every procedure is associated with a failure rate and complications, and the patient has to be aware of these.

In upper limb reconstructive procedures, Gebert *et al.*[9] suggest the use of endoprothesis in smaller tumors, older patients and good soft tissue coverage. In the case of a large diaphyseal defect, young patients and poor soft tissue coverage, biological reconstruction should be considered. A vascularized graft could be a choice for defects of up to 6 to 8 cm. For longer defects, the reconstruction is susceptible to failure because of the lack of vascularization, and for defects greater than 12 cm, the bone graft is never completely replaced by healthy tissue and remains weaker than normal bone, increasing the risk of fracture.

For longer defects, a vascularized fibula graft (VFG) displays an increased initial graft strength and a more rapid union.

Although no final conclusion could be drawn from one case, we consider, with other authors [10], that, in the case of malignant tumor of the distal ulna, the achievement of good margins after resection is of primary importance. Additional reconstructive procedures impact on the morbidity and add the risk of complications that are not justified by the functional improvement; moreover, they require special technical skills and are not routinely justified for this rare condition. Afterwards, we suggest the preventive synthesis of the radius if partial osteotomy is required when the ulna is resected.

Poor soft tissue coverage is clearly the only indication, according to the authors, for a non-reconstruction technique in a longer resection.

## Consent

Written informed consent was obtained from the patient for publication of this case report and any accompaining images. A copy of the written consent is available for review by the Editor-in-Chief of this journal.

## Abbreviations

CT: computed tomography; MRI: magnetic resonance imaging; PMMA: poly(methyl methacrylate); VFG: vascularized fibula graft.

## Competing interests

The authors declare that they have no competing interests.

## Authors’ contributions

All authors contributed equally to this work.

All authors read and approved the final manuscript.
